# New Treatment Options for Advanced Gastroesophageal Tumours: Mature for the Current Practice?

**DOI:** 10.3390/cancers12020301

**Published:** 2020-01-28

**Authors:** Hannah Christina Puhr, Matthias Preusser, Gerald Prager, Aysegül Ilhan-Mutlu

**Affiliations:** 1 Department of Medicine I, Division of Oncology, Medical University of Vienna, Waehringer Guertel 18-20, A-1090 Vienna, Austria; hannah.puhr@meduniwien.ac.at (H.C.P.); matthias.preusser@meduniwien.ac.at (M.P.); gerald.prager@meduniwien.ac.at (G.P.); 2 Comprehensive Cancer Center Vienna—Gastroesophageal Tumors Unit, Waehringer Guertel 18-20, A-1090 Vienna, Austria

**Keywords:** gastric cancer, gastroesophageal cancer, targeted therapy, chemotherapy, immunotherapy

## Abstract

Several clinical trials attempted to identify novel treatment options for advanced gastroesophageal tumours in first, second and further lines. Although results of targeted therapy regimens were mainly disappointing, novel immunotherapy agents showed promising activity, which led to their approval in second and third lines in many countries. This review focuses on the results of recent clinical trials investigating novel agents including targeted therapies, immunotherapy components and chemotherapies and discuss their current impact as well as current approval status on the treatment armamentarium of advanced gastroesophageal tumours.

## 1. Introduction and Background

Cancer of the upper gastrointestinal (GI) tract is a frequent disease, particularly in Asian countries, and a major contributor to global disease burden and therefore a widely researched topic [1]. However, the overall survival (OS) and prognosis remain poor especially in more advanced stages. Thus, a multidisciplinary approach and new treatment options are essential for patients with cancer of the upper gastrointestinal tract [2].

Since there are major differences concerning aetiology, incidence as well as treatment options, gastroesophageal cancer may be seen as two individual entities including gastric and gastroesophageal junction cancer (adenocarcinoma) as well as oesophageal cancer (squamous cell carcinoma). In spite of this vast diversity, the prognosis of both entities is similar. The 5-year relative survival for oesophageal and gastric cancer is 25.1% and 31.0% at locally advanced stages, and 4.8% and 5.3% at metastatic stages, respectively [3,4]. Seen as these malignant diseases progress very fast, several treatment lines are required to offer these patients the appropriate and evidence-based therapy as their disease advances.

Recently, there have been several clinical phase III and phase II studies to investigate the benefit of different therapy regimens including immunotherapy, targeted therapy and chemotherapy on the overall survival of patients with gastroesophageal tumours in several lines of treatment. Results of some of these trials have a major impact on the clinical practice and, thus, are discussed in this review of new treatment options for patients with metastatic gastroesophageal cancer.

## 2. Gastric and Gastroesophageal Junction Adenocarcinoma 

### 2.1. Immunotherapy

#### 2.1.1. Third and Further Lines

The first promising results concerning immunotherapy in advanced and metastatic settings were achieved in the clinical phase III ATTRACTION-2 study (ONO-4538-12), investigating the programmed cell death receptor 1 (PD-1) checkpoint inhibitor nivolumab in an Asian cohort (participating countries: Japan, Taiwan and Korea), and the clinical phase II KEYNOTE-059 study, investigating the programmed cell death receptor 1 inhibitor pembrolizumab in a Western cohort (16 participating countries, mainly non-Asian) [5,6]. Although, the patients included into both studies were heavily pre-treated and thus already refractory to at least two treatment regimens, the overall response upon immunotherapy was promising (ATTRACTION-2: median OS 5.26 months in the nivolumab group and 4.14 months in the placebo group; hazard ratio (HR) 0.63, 95% confidence interval (CI) 0.51–0.78; *p* < 0.0001; objective response in the nivolumab group 11.2%; 7.7–1.·6, objective response in the placebo group 0%; 0–2.8; KEYNOTE-059: objective response rate 15.5%; 95% CI: 10.1%–22.4%; 23 of 148 patients). Furthermore, exploratory analysis of the ATTRACTION-2 of programmed cell death receptor ligand 1 (PD-L1) expression status showed that the benefit on the overall survival was independent of the programmed cell death receptor ligand 1 status. In the KEYNOTE-059 trial however, the median response duration was 16.3 (1.6+ to 17.3+) months and 6.9 (2.4 to 7.0+) months in patients with programmed cell death receptor ligand 1−positive and programmed cell death receptor ligand 1−negative tumours, respectively, indicating that the programmed cell death receptor ligand 1 status of the patient is an important prognostic marker for the treatment response [6].

Furthermore, recently published data on the long-term follow up from the KEYNOTE-059 trial demonstrate manageable safety and a superior long-term overall survival (OS of 1 year/2 years was 24.6%/12.5%, 52%/32% and 63.6%/40.1% in cohorts 1, 2 and 3, respectively) [7], thus suggesting that treatment with pembrolizumab confers sustained responses and disease control in patients with advanced gastric or gastroesophageal junction adenocarcinoma.

In the ATTRACTION-2 study the most common adverse events of nivolumab noted were pruritus, diarrhoea, rash and fatigue and there very relatively few (10%) grade 3 or 4 treatment-related adverse events. Thus, the safety profile of nivolumab in patients with advanced gastric or gastro-oesophageal junction cancer was manageable and similar to that reported in patients with other advanced solid tumours treated with anti-programmed cell death receptor 1 antibodies. Similar toxicity profiles were observed in the KEYNOTE-059 trial. The most common adverse events of pembrolizumab were hypothyroidism, hyperthyroidism and colitis and only 4.6% of patients experienced grade 3 or 4 events. Due to the data derived by these studies nivolumab and pembrolizumab were approved as salvage therapies by some Asian authorities (Taiwan, South Korea and Japan) and the FDA, respectively [8,9,10,11].

In addition to these key findings another checkpoint inhibitor avelumab in the JAVELIN-GASTRIC-300 study, was investigated in comparison with physicians’ choice of either irinotecan or paclitaxel as chemotherapy, but failed to demonstrate superior overall survival (OS) with single-agent avelumab (median OS 4.6 versus 5.0 months; HR 1.1, 95% CI 0.9–1.4; *p* = 0.81) [12]. However, avelumab showed a more manageable safety profile than chemotherapy, thus leading to the assumption that this treatment option may be suitable for fragile patients. Further studies are needed to confirm and pursue this strategy.

#### 2.1.2. Second Line

In the KEYNOTE-061 trial pembrolizumab was compared to paclitaxel as a second-line treatment in programmed cell death receptor ligand 1 positive patients, but there was no clinically meaningful survival benefit between the groups (median OS: 9.1 months (95% CI 6.2–10.7) with pembrolizumab and 8.3 months (7.6–9.0) with paclitaxel; HR 0.82, 95% CI 0.66–1.03; one-sided *p* = 0.0421) [13]. However, a post hoc analysis of this study showed a survival benefit for patients with microsatellite instability (MSI) high (MSI-H) tumours as well as tumours with combined positive score (CPS) >10. Thus, surmising that microsatellite instability is a valuable predictive marker for the response to immunotherapy in addition to programmed cell death receptor ligand 1 expression. Consistently, this trial demonstrated a better safety profile of immunotherapy compared to chemotherapy.

Thus, additional trials of pembrolizumab in gastric and gastro-oesophageal cancer after failure of chemotherapy are ongoing and need to evaluate the efficacy of this treatment option in preselected patient subgroups. Furthermore, in the KEYNOTE-061 trial the control group received chemotherapy with paclitaxel, which is not the current standard of care for second line treatment. Hence, further trials are needed to draw comparisons between immunotherapy and current second line treatment options.

#### 2.1.3. First Line

Since the results of nivolumab and pembrolizumab in the late line of treatment were promising, these agents are tested in a first line setting in combination with standard chemotherapy regimens in the still ongoing CHECKMATE-649 and the KEYNOTE-062 study, respectively [14,15].

In the open-label, phase 3 CHECKMATE-649 trial the combination of the programmed cell death receptor 1 checkpoint inhibitor nivolumab and the cytotoxic T-lymphocyte-associated protein 4 inhibitor ipilimumab will also be investigated. Thus, the results of this study might bring a major breakthrough in the field of first line combined immunotherapy in metastatic cancer of the upper gastrointestinal tract. Furthermore, avelumab is analysed as a maintenance treatment strategy in the JAVELIN-GASTRIC-100 study.

A recent phase II KEYNOTE-059 study (cohort II and III) demonstrated the anti-tumour activity and moderate toxicity profiles of pembrolizumab as monotherapy and in combination with chemotherapy in a first line setting (objective response rate: 25.8% (95% CI 11.9–44.6) and 60.0% (95% CI, 38.7–78.9), respectively) [16]. Until then, there had been no evaluation of a potential benefit upon a combination strategy of immunotherapy plus chemotherapy when compared to chemotherapy alone. The median overall survival of patients treated with the combination of chemotherapy plus pembrolizumab was 13.8 months (95% CI 8.6–not estimable) compared to 20.7 months (95% CI 9.2–20.7) with pembrolizumab monotherapy [16].

At ASCO Annual Meeting 2019, the first results of the clinical phase III KEYNOTE-062 study were presented, which demonstrated pembrolizumab was non-inferior to chemotherapy alone for overall survival in combined positive score ≥1 tumours. A superior and clinically meaningful benefit by improvement of OS, however, was seen in patients with high programmed cell death receptor ligand 1 expressing tumours of combined positive score ≥10 (median OS 17.4 vs. 10.8 months; HR 0.69, 95% CI 0.49–0.97), but this was not analysed for statistical significance due to the study’s hierarchical design. Surprisingly, the combination of the checkpoint inhibitor with chemotherapy was not superior for OS, neither in combined positive score ≥1 nor in combined positive score ≥10 tumours when compared to chemotherapy alone, but showed a favourable trend for the combination (median OS in patients with CPS ≥ 10: with combination immunochemotherapy 12.3 vs. 10.8 months with chemotherapy; HR 0.85, 95% CI 0.62–1.17; *p* = 0.158) [15]. As observed in several other immune checkpoint inhibition trials, the rates of serious side effects were lowest among patients treated with pembrolizumab alone. Surprisingly, the patients’ reported quality of life data using 2 different assessments (EORTC QLQ-C30 and EORTC QLQ-STO22 scales) did not show any improvement of quality of life in the cohort of patients with pembrolizumab alone against chemotherapy [17].

Although the Kaplan–Meier survival curves insinuate that (combination) immunotherapy even has a worse survival rate in the first few months of administration compared to chemotherapy, the curves then cross each other and (combination) immunotherapy shows promising results in the long-term treatment. These suspenseful results might surmise that special subgroups of patients might have a greater benefit from immunotherapy than others and that the identification of these patients might be a great challenge for further studies.

In this regard an exploratory analysis of the KEYNOTE-062 study presented at the ESMO Annual Meeting 2019 indicated that patients with microsatellite instability high and combined positive score ≥1 tumours might benefit from pembrolizumab +/− chemotherapy compared to chemotherapy alone (pembrolizumab vs. chemotherapy: median OS not reached vs. 8.5 months; HR 0.29; 95% CI 0.11–0.81 and pembrolizumab + chemotherapy vs. chemotherapy: Median OS not reached vs. 8.5 months; HR 0.37; 95% CI 0.14–0.97) [18]. Interestingly, the previous results stating the non-inferiority of pembrolizumab monotherapy against chemotherapy in combined positive score ≥1 patients as well as the superior outcome of pembrolizumab monotherapy against chemotherapy in combined positive score ≥10 patients remain significant, when microsatellite instability high patients are excluded from the cohort, suggesting that the presence of microsatellite instability high patients did not substantially affect the outcome of this clinical trial.

However, despite these promising findings, it is important to await the final results of the KEYNOTE-062 trial before recommending pembrolizumab in a first line setting in order to provide a safe and sufficient treatment option.

### 2.2. Targeted Therapies

#### 2.2.1. Third and Further Lines

Apatinib (Rivoceranib), a VEGFR2 tyrosine kinase inhibitor, showed positive results compared to placebo in a Chinese population, leading to its approval by Chinese authorities [19]. The phase III trial ANGEL is currently studying apatinib in a Western cohort, but first results, which were presented at the ESMO Annual Meeting 2019 [20], were disappointing. The overall survival was not significantly improved in the overall population, while a subgroup analysis demonstrated a benefit for patients in a ≥4th line setting as well as patients with liver metastasis. Apatinib also seems to have a favourable tolerability profile. However, these findings failed to shift the paradigm of further line therapy strategies in the Western population.

Regorafenib, a tyrosine kinase inhibitor targeting multiple angiogenesis and growth pathways, is currently under investigation in the phase III INTEGRATE-II trial [21]. The final results of these studies are not yet published. Furthermore, the phase Ib REGONIVO trial investigated the combination therapy of regorafenib and nivolumab and showed promising results concerning manageable safety profiles and encouraging anti-tumour activity [22]. Thus, further investigations in a larger cohort are planned.

#### 2.2.2. Second Line

In the second line setting, the results of the REGARD and RAINBOW studies, which investigated the effect of ramucirumab, an antibody against VEGFR2, alone (median OS with ramucirumab 5.2 months vs. 3.8 months in the placebo group; HR 0.776, 95% CI 0.603–0.998; *p* = 0.047) and in combination with paclitaxel (median OS with paclitaxel and ramucirumab 9.6 months vs. with paclitaxel 7.4 months; HR 0.807, 95% CI 0.678–0.962; *p* = 0·017) have to be considered [23,24]. Both studies showed positive results and consequently leading to the incorporation of ramucirumab as a favourable treatment option in second line in European as well as U.S. guidelines [25,26]. Thus, also patients who are not fit for second line chemotherapy might have a benefit, if treated with ramucirumab as a monotherapy. These promising results were affirmed by the “real-life” efficacy data on ramucirumab treatment in the RAMoss study (median OS 8.0 months; 95% CI 7.09–8.9) [27].

Other promising targeted therapy options did not show a significant benefit in the OS, e.g., the BRIGHTER study which investigated the inhibition of STAT3 with napabucasin [28].

#### 2.2.3. First Line

The last decade brought us several studies investigating various targeted therapies in a first line setting. Regrettably, none of the results showed a significant benefit for patients with gastric and gastroesophageal junction cancer. However, several studies with negative results are mentionable: EGFR inhibition with cetuximab (EXPAND study) and with panitumumab (REAL-3 study), MET/HGF inhibitors rilotumumab (RILOMET-1) and Onartuzumab (METGastric study) as well as angiogenesis inhibition with bevacizumab (AVAGAST and AVATAR studies) and with ramucirumab (RAINFALL study) [29,30,31,32,33,34,35].

The most recent results targeting the microenvironment concerning the MMP-9 inhibitor andecaliximab (phase III GAMMA-1 trial) were presented at the ASCO GI Meeting 2019, but failed to show a clinical benefit. Although the response rates were significantly better in the andecaliximab group than in the placebo group (response rates: 50.5% vs. 41.1%; complete responses: 8.3% vs. 4.7%, *p* = 0.049), there was no difference in the median overall survival (12.5 vs. 11.8 months; HR 0.93; *p* = 0.56) [36].

A ray of hope on the horizon of targeted therapies in a first line setting is provided by the phase II FAST study, which showed a sufficient inhibition of the tight junction protein claudin 18.2 with the monoclonal antibody zolbetuximab (median OS 8.4 months with chemotherapy regimen EOX vs. 13.2 months with zolbetuximab; HR 0.51, 95% CI 0.36–0.73; *p* = 0.0001) [37]. Due to this finding, two large phase III trials investigating zolbetuximab with different chemotherapy backbones, either with CAPOX or FOLFOX, are currently ongoing and results are expected to be published in the next few years.

### 2.3. Chemotherapy

#### 2.3.1. Third and Further Lines

The combination of the chemotherapeutic agents trifluridine and tipiracil, which is already well established in colorectal cancer as third and further line treatment, was recently investigated in patients with advanced and metastatic gastric and gastroesophageal junction cancer in the TAGS study [38]. This novel oral cytotoxic chemotherapy has a unique mechanism of action in which Trifluridine is incorporated into DNA, resulting in DNA dysfunction, and tipiracil blocks trifluridine degradation by thymidine phosphorylase, increasing trifluridine bioavailability. Compared to placebo this combination therapy showed an improvement in overall survival (5.7 months in the trifluridine/tipiracil group versus 3.6 months in the placebo group; HR 0.69, 95% CI 0.56–0.85; *p* = 0.00058). The common adverse events were haematological side effects, which could be managed with routine procedures including G-CSF support, transfusions or dose reductions. As a rational concern for an oral drug for bioavailability, a post-hoc analysis for patients with gastrectomy was recently demonstrated, where the survival advantage in the trifluridine and tipiracil arm remained significant [39]. Quality of life data also indicates an improvement of cancer-related symptoms within the trifluridine/tipiracil group [40]. Thus, the U. S. Food and Drug Administration (FDA) and European Medicines Agency (EMA) approved this regimen as salvage treatment for patients with metastatic or advanced gastroesophageal adenocarcinoma, whose tumour progressed after 2 treatment regimens [41].

#### 2.3.2. Second Line

Besides from targeted therapy agents, a taxane therapy, usually paclitaxel represents a common treatment choice in a second line setting. A large phase III trial (ABSOLUTE trial) showed the noninferiority of weekly nab-paclitaxel to paclitaxel, which is an important finding considering the lower toxic and better pharmacokinetic properties of the albumin-bound agent. This led to the approval of nab-paclitaxel as a second line treatment option in Japan [42,43].

Furthermore, the findings of the COUGAR-02 trial suggest that docetaxel can be recommended as an appropriate second-line treatment for patients who are refractory to treatment with platinum and fluoropyrimidine (median OS with docetaxel 5.2 months versus 3.6 months in the active symptom control group; HR 0.67; 95% CI 0.49–0.92; *p* = 0.01) [44].

Aside from that, irinotecan might also be a valid second line option as demonstrated in the WJOG 4007 trial (median OS was 9.5 months in the paclitaxel group and 8.4 months in the irinotecan group; HR 1.13, 95% CI 0.86–1.49; *p* = 0.38) [45].

#### 2.3.3. First Line

There were no approvals of new chemotherapeutic agents in a first line setting in the last few years. However, the chemotherapy regimen FLOT (combination of leucovarin, 5-FU, oxaliplatin and docetaxel) represents a quite novel treatment regimen, which showed promising results in a first line perioperative setting (FLOT4 trial, median OS 50 months (38·33 to not reached) in FLOT group vs. 35 months in standard chemotherapy group; HR 0.77; 95% CI 0.63–0.94) [46]. Thus, this relatively new regimen might also be considered as first line therapy for patients with an excellent performance status in metastatic settings.

However, it is important to consider, that this regimen was investigated in resectable tumours, with no evidence of distant metastases, and currently there is no available data for advanced settings. There is no properly designed phase III trial testing the doublet treatment regimen against a triplet in metastatic tumours [47]. However, retrospective analyses in Spain and Italy suggested that triplet therapy might be associated with a discreet benefit in efficacy at the expense of an increase in toxicity [48,49]. A recently published systematic review and meta-analysis also stated that compared with doublet chemotherapy, triplet chemotherapy improved the overall survival in patients with advanced gastric cancer [50]. But a recent Dutch retrospective survey of this chemotherapy regimen used in a first line palliative setting demonstrated no survival benefit in triplet chemotherapy patients compared to doublets [51].

Furthermore, whether gastrectomy in addition to chemotherapy in a first line setting might benefit the overall survival is a highly researched topic. The findings of the REGATTA trial demonstrated that gastrectomy followed by chemotherapy did not show any survival benefit compared with chemotherapy alone in patients with a single non-curable factor, thus concluding that initial gastrectomy cannot be justified as a treatment option [52]. However, the randomized phase III trial RENAISSANCE (AIO-FLOT5) is currently investigating whether oligo-metastatic patients with an excellent performance status have a benefit from gastrectomy with perioperative chemotherapy with the FLOT regimen [53]. Patients without disease progression after 4 cycles are randomized 1:1 to receive additional chemotherapy cycles or surgical resection of primary and metastases followed by subsequent chemotherapy. If this concept proves to be effective, this could potentially lead to a new treatment option for patients with an oligo-metastatic disease and an excellent performance score.

## 3. Oesophageal Cancer

The most recent breakthrough concerning advanced and metastatic oesophageal cancer was the presentation of the findings of the KEYNOTE-181 trial at the ASCO GI Meeting 2019. Patients with oesophageal tumours, adenocarcinomas and squamous cell carcinomas, with a combined positive score of 10 or more, which accounted for about 35% of the study population, benefited from pembrolizumab in a second line setting when compared to chemotherapy (median OS of 9.3 months with pembrolizumab vs. 6.7 months with chemotherapy; HR 0.69, *p* = 0.0074). The 12-month survival rate in this group was 43% vs. 20%, respectively. Nevertheless, it did not improve overall survival or progression-free survival in the overall intent-to-treat population, but a trend was observed favouring pembrolizumab in patients with squamous cell carcinoma [54]. These differences favouring pembrolizumab did not meet the study’s prespecified statistical boundary of *p* ≤ 0.0075. The U. S. Food and Drug Administration approved this treatment in patients with squamous cell carcinoma and a combined positive score ≥10.

Almost simultaneously, first findings of the CHECKMATE-473/ATTRACTION-3 trial were published, that showed a survival benefit in a second line setting in patients with oesophageal squamous cell carcinomas [55]. The median overall survival in patients treated with nivolumab compared to chemotherapy with docetaxel or paclitaxel was improved independent of programmed cell death receptor ligand 1 expression (median OS: 10.9 months vs. 8.4 months; HR 0.77, 95% CI 0.62–0.96; *p* = 0.019). The most frequent grade 3 or 4 treatment-related adverse events were haematological side effects, anaemia in the nivolumab and leukopenia in the chemotherapy group and the incidence of treatment-related adverse events leading to discontinuation was similar in both groups. However, nivolumab showed a statistically significant overall improvement in quality of life versus chemotherapy [56]. An important fact to mention is that 401 (96%) of 419 enrolled patients were Asian, which might limit the interpretation of the results for non-Asian patients. However, an analysis in Asian and non-Asian patients showed that the overall survival favoured nivolumab in both subgroups.

Furthermore, the identification of other subgroups might also be important in this entity to improve patient selection for new treatment options. In this study the Kaplan–Meier curves for the overall survival also crossed, so it can be surmised that specific subgroups might have a greater benefit from the immunotherapy and should be investigated in further studies.

In first line setting there are several ongoing trials evaluating the efficacy of immunotherapies, especially the results of the phase III trials KEYNOTE-590 (comparing standard chemotherapy with and without pembrolizumab) and the CHECKMATE-648 (comparing chemotherapy alone, combination with nivolumab and combination with nivolumab and ipilimumab) should bring new insights in the treatment of oesophageal squamous cell carcinoma [57,58].

## 4. Her2 Positive Gastroesophageal Tumours

Her2 positive tumours of the upper gastrointestinal tract are a widely researched topic. Since the approval of the monoclonal antibody trastuzumab against Her2 in 2010 following the TOGA trial, a first effective targeted therapy is available for this subgroup of patients [59]. Unfortunately, all recent studies following this major breakthrough did not show any positive results in any treatment lines.

The inhibition of Her2 was the focus of the LOGIC study using lapatinib (median OS with lapatinib 12.2 and with placebo 10.5 months; HR 0.91, 95% CI 0.73–1.12) and the JACOB study using a combination treatment of trastuzumuab plus pertuzumab with chemotherapy in a first line setting (median OS 17.5 months in the pertuzumab group and 14.2 months in the control group; HR 0.84, 95% CI 0.71–1.00; *p* = 0.057) [60,61].

As a second line option after disease progression while administering trastuzumab, T-DM1 was investigated in a large phase III trial, but failed to improve the overall survival while causing a similar amount of adverse events to standard chemotherapy (GATSBY trial; median OS: 7.9 months with trastuzumab emtansine and 8.6 months with taxane treatment; HR 1.15, 95% CI 0.87–1.51, one-sided *p* = 0.86) [62]. Another phase III trial, the TyTAN trial, investigated the Her2 inhibitor lapatinib plus paclitaxel versus paclitaxel alone in a second line setting in an Asian population with Her2 positive advanced gastric cancer. Although the addition of lapatinib demonstrated activity in the second-line treatment, it did not significantly improve the overall survival in the intent-to-treat population (11.0 months with lapatinib plus paclitaxel versus 8.9 months with paclitaxel alone; HR 0.84; 95% CI 0.64–1.11; *p* = 0.104) [63].

Another question that arose with the paradigm shifting results of the TOGA trial, was whether trastuzumab should be administered beyond progression. Results of a phase II study comparing the combination of paclitaxel with beyond progression trastuzumab versus paclitaxel alone in Her2 positive patients, who already received first line antibody/chemotherapy combination therapy, showed that there was no benefit of combination therapy concerning the overall survival (9.95 months with paclitaxel versus 10.2 months in the paclitaxel + trastuzumab arm; HR = 1.23; 95% CI 0.75–1.99; *p* = 0.20) [64]. It is surmised that this effect might be due to the loss of HER2 after first line treatment, since almost 2/3 of patients lost their Her2 positivity, which was evaluated in an additional biopsy at the time of progression under trastuzumab [64,65]. However, some new retrospective analyses suggest that although there is no improvement of the OS, patients might still have a clinical benefit from the continuation of Trastuzumab beyond first-line progression [66,67]. These results deserve a prospective randomized validation.

The combination of the immunotherapy agent pembrolizumab with the TOGA protocol has recently been investigated in Her2 positive metastatic or advanced gastroesophageal adenocarcinoma patients in a phase II trial. Notably, all patients had a tumour regression ranging from −4% to −100% (RECIST 1.1 objective response rate was 89% with 27 partial remissions and 3 complete remissions) and a median overall survival of 27 months, which is significantly higher than in the historical control group of the TOGA trial [68,69]. Furthermore, this outcome was programmed cell death receptor ligand 1 independent. These promising findings will be further investigated in the phase III KEYNOTE-811 trial [70].

Furthermore, the mechanisms of primary resistance against anti-Her2-treatment is of high clinical interest. It is surmised that resistant tumours have activated other tyrosine kinase receptors or downstream signalling pathways. To avoid unnecessary toxicity of the therapy with no clinical benefit, it is a crucial task to determine which tumours are resistant before initiating therapy. However, although several genomic alterations are suspected to be clinically useful to predict primary resistance to Trastuzumab in HER2-positive metastatic gastric cancer patients, none of them have yet been included into routine evaluations [71].

## 5. Tissue Agnostic Approval and Cancer Genome Atlas

In the last few years, due to novel drug developments such as immunotherapy or targeted therapy, new strategies to target cancer cells have been introduced to modern oncology practice. In drugs with tissue agnostic approval, the treatment of the cancer cells is based on the mutations that they display, instead of the tissue type in which they appear. This novel strategy led to new treatment options also in gastroesophageal cancer patients.

Recently, advances in technology and high-throughput analysis have also improved our understanding of the genetic background of gastric cancer. To provide a roadmap for patient stratification and trials of targeted therapies and immunotherapies, the Cancer Genome Atlas (TCGA) Research Network has characterized almost 300 primary gastric adenocarcinomas and proposed a new classification of four different tumour subtypes: Epstein-Barr virus positive, microsatellite instability, genomically stable and chromosomal instability [72]. Based on this new classification tissue agnostic approved therapies might be easily applicable in gastric cancer patients.

### 5.1. Microsatellite Instability

In 2017 the U.S. Food and Drug Administration approved pembrolizumab for the treatment of unresectable or metastatic, microsatellite instability high/mismatch repair deficiency solid tumours, which have no satisfactory alternative treatment options after at least one prior treatment regimen [73]. Based on this “tissue agnostic approval”, testing of microsatellite instability high/mismatch repair deficiency in tissues of all tumour types might have a therapeutic consequence and should therefore be considered if alternative treatment strategies are not satisfactory.

The clinical and pathological characteristics of microsatellite instability are a widely researched topic in gastric cancer patients. Thus, many studies were conducted to enhance the knowledge on this particular matter and in large part these studies demonstrated an association of high microsatellite instability with age, female gender, distal third of the stomach, intestinal pathology, lower pTNM stage and lower number of infiltrated lymphnodes. It is assumed, that approximately one quarter of all Western gastric cancer cases is microsatellite instability high [74,75,76,77,78]. Distinct genetic features including increased number of tumour infiltrating lymphocytes and programmed cell death receptor ligand 1 positivity is generally associated with this subgroup of gastric tumours [74,78].

In this regard, a post-hoc analysis of the British MAGIC trial, which represents a major breakthrough in the treatment of resectable tumours by demonstrating a survival benefit when treated perioperatively with the chemotherapy combination Epirubicin, Oxaliplatin and Capecitabine, was conducted [79]. The tissue samples were re-analysed and results showed, that patients with microsatellite instability high tumours benefited less from chemotherapy than the rest of the population. Thus, it was surmised that other treatment strategies should be considered for this selected patient group [80].

Additionally, the post-hoc investigation of the CLASSIC trial affirmed these results and led to similar conclusions. In this Asian study patients were randomized to adjuvant chemotherapy or surgery alone and the post-hoc analysis also failed to show a survival benefit for microsatellite instability high patients, when treated with post-operative chemotherapy [81].

Furthermore, a recently published meta-analysis of the value of microsatellite instability as a biomarker in gastric cancer suggested it to be a robust prognostic marker that should be adopted as a stratification factor by clinical trials and investigated prospectively [82]. Thus, there currently are several randomized prospective trials with microsatellite instability high patients, treated with surgery only in a resectable setting. These investigations are surmised to clarify, whether we should shift the current treatment paradigm in resectable settings and treat this patient subgroup solely with gastrectomy or with alternative therapies, such as immunotherapy, instead of conventional chemotherapy.

### 5.2. Epstein-Barr Virus

Tumour Epstein-Barr Virus (EBV) positivity is suspected to be a major driver in some cancer entities. In the last few years there has been extensive research on the viral status in gastric cancer patients and Epstein-Barr Virus (EBV) positivity is an emerging marker, which might be introduced in personalized treatment. The positivity rate among patients with upper gastrointestinal cancer is estimated to be around 8%–10% and, unlike in other entities, the distribution of Epstein-Barr Virus-associated gastric carcinoma worldwide is surmised to be approximately even [83]. So far, the precise role of the Epstein-Barr Virus as a prognostic factor is not fully clarified, however, several studies indicate the association of viral positivity with a longer overall survival [84,85,86]. Additional investigations demonstrate an enhancement of programmed cell death receptor ligand 1 expression in Epstein-Barr Virus-positive gastroesophageal tumours. This association suggests this subgroup might benefit from immunotherapy targeting the programmed cell death receptor ligand 1/programmed cell death receptor 1 axis [87,88].

Emphasizing this assumption, a phase II biomarker trial, conducted on a Epstein-Barr Virus positive cohort, recently showed a 100% overall response rate when treated with pembrolizumab after one prior treatment line [89]. However, so far large clinical trials to confirm these results are missing and thus, the investigation of Epstein-Barr Virus is not routinely recommended in international guidelines. Nevertheless, many academic hospitals have already implemented testing for Epstein-Barr Virus as a part of routine diagnostics and therefore this marker might play an important role in upcoming studies.

## 6. Discussion and Conclusions

Even though gastroesophageal tumours are a major contributor to global disease burden and therefore a widely researched entity, the OS, especially in advanced and metastatic stages, is still poor [3,4]. Due to the localization of the primary tumour symptoms of advanced stages often include tumour-specific symptoms such as dysphagia, dyspepsia, vomiting, early satiety and/or iron deficiency anaemia, additionally to common symptoms of malignant diseases like weight loss and fatigue, thus, adding to the burden of the cancer itself and leading to reduced quality of life [90].

New treatment options are therefore desperately needed to improve the survival as well as the quality of life of this large number of patients worldwide. Especially in second, third and further line settings evidence from recent clinical phase 3 trials have emphasised the importance of multiple lines of therapeutic options [91,92]. This review illustrates the variety of studies conducted in the last few years to improve therapeutic strategies in patients with advanced and metastatic cancer of the upper gastrointestinal tract in multiple treatment lines. The Table 1 and Table 2 give an overview of recent trials discussed in this review as well as recent and expected approvals of second as well as third and further line therapy strategies in locally advanced and metastatic settings, respectively.

In the last decade there are numerous studies with promising study designs, especially considering immunotherapies and targeted therapies. Since inhibition of checkpoints changed the treatment paradigm in various of tumour types including those derived from lung, kidney or oropharynx, it was surmised that gastroesophageal cancer patients might also have a benefit from this new treatment option. However, in large part trials with checkpoint inhibitors failed to meet these high expectations. Only under certain clinical conditions immunotherapy agents have demonstrated to provide an improved prognosis when compared to standard care, and led to approval by some authorities. These drugs as well as their approval status can be seen in Table 2. 

The ATTRACTION-2 and the KEYNOTE-059 trials provide robust evidence for the use of nivolumab and pembrolizumab, respectively, in a third-line setting for appropriate patients [5,6]. Thus, these drugs were already approved by some Asian and American authorities, respectively. However, both studies evaluated immunotherapy in salvage setting and there was a lack of a control group in the KEYNOTE-059 trial, whereas the ATTRACTION-2 trial only had treatment with placebo as a control arm. These results are a major breakthrough considering new treatment options, but have to be evaluated in comparison to other drugs, particularly with regard to new chemotherapeutics, which also prolong the overall survival with a reasonable amount of adverse events. The combination chemotherapy with trifluridine and tipiracil was evaluated as a third line treatment in the TAGS trial, which led to the drug approval in this setting in the United States of America as well as in Europe due to the significant improvement of overall survival compared with placebo. Since this combination chemotherapy is administered orally, the quality of life of patients might benefit in the sense that they do not have to be hospitalized for the treatment itself [38]. Nevertheless, emphasis has to be placed on the fact, that this new chemotherapy regimen was also evaluated in a placebo-controlled trial.

Another factor to consider when determining whether targeted therapy, immunotherapy or chemotherapy is more appropriate in advanced gastroesophageal cancer is the individual’s tumour profile. Therefore, the proposed new classification of gastric cancer subtypes by the TCGA might play an important role in further clinical trials and consequently new treatment decisions. Although nivolumab was approved in a third line setting in Asia regardless of the molecular profile and combined positive score, there are several findings in other studies, which emphasise different responses in different tumour subgroups. In the KEYNOTE-181 trial pembrolizumab was most effective in the subgroup with combined positive score ≥10 and a post hoc analysis of the KEYNOTE-061 trial showed that microsatellite instability high tumours showed the most promising response to checkpoint-inhibition [13,54]. These results are strengthened by the findings of the KEYNOTE-062 trial that showed the greatest benefit and clinically meaningful improvement of overall survival in patients with high expression of programmed cell death receptor ligand 1 with a combined positive score ≥10. Furthermore, the Kaplan–Meier survival curves of the KEYNOTE-062 trial insinuate that (combination) immunotherapy in some patients even has a worse survival rate in the first few months of administration compared to chemotherapy, the curves then cross each other and (combination) immunotherapy shows promising results in the long term. Thus, new treatment schedules with first 3–6 months chemotherapy followed by immunotherapy maintenance might pose as reasonable treatment options to improve the overall survival and therefore most likely will be tested in further trials.

Also, it is surmised, that some targeted therapies might have a better benefit in an Asian population due to the already demonstrated exhibition of distinct gene-expression signatures related to T-cell function compared with non-Asian patients [93]. Different genetic and molecular subtypes in different populations might also be the reason why apatinib (rivoceranib) is an established and approved third line therapy in China, but failed to provide satisfactory results in a Western cohort within the ANGEL trial [20]. Thus, a better understanding and more insight in the molecular profiling of cancer subgroups, might also provide us with better treatment strategies for individual patients.

Other trials, discussed in this review, were conducted to compare immunotherapy to standard treatment options in second or further line settings and found no significant difference in the median OS [12,13]. However, immunotherapy and targeted therapy might still be valid treatment options compared with standard chemotherapy in a second line setting due to their favourable safety and toxicity profiles. In the KEYNOTE-061 trial, for example, there was no benefit in the overall survival for patients treated with pembrolizumab compared to standard treatment with Paclitaxel, however, grade 3–5 treatment-related adverse events occurred in only 14% of the patients treated with the checkpoint-inhibitor and in 35% of the patients treated with chemotherapy [13]. The KEYNOTE-062 trial demonstrated the favourable safety profile of pembrolizumab compared to chemotherapy. Grade 3–5 drug-related adverse event rates were 17% with pembrolizumab, 73% with immunochemotherapy and 69% chemotherapy alone [18]. Hence, the toxicity profile of therapies has to be considered and discussed with the patient when deciding which therapy is the best, particularly for the patients with biomarker positivity. It was, however, surprising to see that the documented better toxicity profile of pembrolizumab was not translated into an improved quality of life in patients with pembrolizumab monotherapy when compared to chemotherapy [17]. Further subgroup analysis and long-term results of this data are however lacking. It is therefore important to integrate the quality of life assessments into clinical trials, particularly those questionnaires that focus on the specific symptoms and problems of gastroesophageal tumour patients.

Not only new adverse events, but also residual toxicity from prior therapies, for example neutropenia and polyneuropathy, have to be taken into consideration before a new treatment strategy can be fixed. Comorbidities such as renal and hepatic impairment might also limit therapeutic options. Thus, study designs to show non-inferiority of new agents are therefore a solid way to evaluate better toxicity profiles and to expand treatment strategies and improve the quality of life for patients with metastatic cancer of the upper gastrointestinal tract. The ABSOLUTE study was powered for non-inferiority and could show the advantages of the nab-paclitaxel formulation considering adverse events as well as pharmacokinetics, thus leading to its approval in Japan [42]. Currently, several treatment regimens including nivolumab, pembrolizumab, trifluradine/tipiracil and apatinib are proven to be effective in salvage settings. In order to define an appropriate treatment algorithm for the individual patients, such non-inferiority trials will most probably gain more importance in the future.

Another important issue when discussing novel treatment options is financial toxicity. The costs of new treatment options might exceed the patient’s or health-care system’s limits. Consequently, new drugs which are non-inferior to already established (and therefore most of the time significantly cheaper) treatment options might not find their way into everyday practice. Thus, it is important for clinical trials to evaluate the real-life benefit of novel drug regimens, so that the financial toxicity can be related to the benefits for the patient. The ESMO—Magnitude of Clinical Benefit Scale (ESMO-MCSBS) or ASCO Value Framework Net Health Benefit Score are such tools to stratify the magnitude of clinical benefit that can be anticipated from anticancer therapies [94,95]. Clinical benefit scales should be evaluated in further clinical trials and also involved in individual decision making to optimize patient care and minimize financial toxicity without benefit.

For now, clinical experience from recent studies in gastroesophageal cancer patients but also in other entities might help decide on the best treatment strategies for patients with gastroesophageal cancer. New treatment options, that were approved in the last few years, as well as new study designs might lead to a longer overall survival as well as a better quality of life of patients with upper gastrointestinal cancer in the future. Although, there have been great advances in the diagnosis, understanding and treatment in the last decade, new therapeutic options are still in their infancy. Further studies are necessary to optimize patient care.

## Figures and Tables

**Table 1:** Overview of recent trials discussed in this review.

Name	Trial Number (ClinicalTrials.gov Identifier or Other)	Tumor Type	Setting (Line)	Phase	Population	Treatment Arms	Status	Results (First) Posted
ATTRACTION-2	NCT02267343	Gastric + GEJ * adenocarcinoma	≥3	III	Asia	Nivolumab/Placebo	Active, not recruiting	2 December 2017
KEYNOTE-059	NCT02335411	Gastric + GEJ adenocarcinoma, cohort 3: CPS ≥ 1	1–≥3	II	North and South America, Asia, Europe	Cohort 1: 3rd line, Pembrolizumab Cohort 2: 1st line, Cisplatin + 5-FU + Pembrolizumab Cohort 3: 1st line, Pembrolizumab	Active, not recruiting	10 May 2018
JAVELIN-GASTRIC-300	NCT02625623	Gastric + GEJ adenocarcinoma	≥3	III	North and South America, Asia, Europe	Avelumab/chemotherapy (physician’s choice)	Active, not recruiting	1 October 2018
KEYNOTE-061	NCT02370498	Gastric + GEJ adenocarcinoma	2	III	North and South America, Asia, Europe	Pembrolizumab/Paclitaxel	Active, not recruiting	20 November 2018
CHECKMATE-649	NCT02872116	Gastric + GEJ adenocarcinoma	1	III	Unknown	Cohort 1: Ipilimumab + Nivolumab Cohort 2: Nivolumab + Chemotherapy Cohort 3: Chemotherapy (investigator’s choice)	Active, not recruiting	Estimated May 2021
KEYNOTE-062	NCT02494583	Gastric + GEJ adenocarcinoma, CPS ≥1, HER2-negative	1	III	North and South America, Asia, Europe	Cohort 1: Pembrolizumab Cohort 2: Pembrolizumab + Cisplatin + 5-FU/Capecitabine Cohort 3: Placebo + Cisplatin + 5-FU/Capecitabine	Active, not recruiting	1 June 2019
JAVELIN-GASTRIC-100	NCT02625610	Gastric + GEJ adenocarcinoma, HER2-negative	1	III	North America, Asia, Europe, Australia	Avelumab maintenance/chemotherapy	Active, not recruiting	Estimated November 2019
ANGEL	NCT03042611	Gastric + GEJ adenocarcinoma	≥3	III	North America, Asia, Europe	Apatinib/Placebo	Active, not recruiting	29 September 2019
INTEGRATE-2	NCT02773524	Gastric + GEJ adenocarcinoma or undifferentiated carcinoma	≥3	III	North America, Asia, Australia	Regorafenib/Placebo	Recruiting	Estimated July 2020
REGONIVO	NCT03406871	Gastric, colorectal or hepatocellular cancer	≥2	Ib	Asia	Regorafenib + Nivolumab	Active, not recruiting	2 July 2019
REGARD	NCT00917384	Gastric + GEJ adenocarcinoma	2	III	North and South America, Asia, Europe, Australia	Ramucirumab/Placebo	Completed	4 January 2014
RAINBOW	NCT01170663	Gastric + GEJ adenocarcinoma	2	III	North and South America, Europe, Asia, Australia	Paclitaxel + Ramucirumab/Paclitaxel + Placebo	Completed	1 October 2014
BRIGHTER	NCT02178956	Gastric + GEJ adenocarcinoma	2	III	North America, Asia, Europe, Australia	Napabucasin + Paclitaxel/Placebo + Paclitaxel	Completed	20 May 2018
EXPAND	EudraCT: 2007-004219-75	Gastric + GEJ adenocarcinoma	1	III	Europe	Standard chemotherapy + Cetuximab/standard chemotherapy (investigator’s choice)	Completed	15 April 2013
REAL-3	NCT00824785	Oesophageal + gastric + GEJ adenocarcinoma or undifferentiated carcinoma	1	III	Europe	Epirubicin + Oxaliplatin + Capecitabine + Panitumumab/Epirubicin + Oxaliplatin + Capecitabine	Terminated (Lack of efficacy)	June, 2013
RILOMET-1	NCT01697072	Gastric + GEJ adenocarcinoma, MET-positive	1	III	North and South America, Europe, Asia	Epirubicin + Cisplatin + Capecitabine + Rilotumumab/Epirubicin + Cisplatin + Capecitabine + Placebo	Terminated	November, 2017
METGastric	NCT01662869	Gastric + GEJ adenocarcinoma, HER2-negative, MET-positive	1	III	North America, Europe, Asia	5-FU + Folinic acid + Oxaliplatin + Onartuzumab/5-FU + Folinic acid + Oxaliplatin + Placebo	Completed	1 May 2017
AVAGAST	NCT00548548	Gastric + GEJ adenocarcinoma	1	III	North America, Europe, Asia	Cisplatin + 5-FU or Capecitabine + Bevacizumab/Cisplatin + 5-FU or Capecitabine + Placebo	Completed	22 September 2016
AVATAR	NCT00887822	Gastric + GEJ adenocarcinoma	1	III	Asia	Cisplatin + 5-FU or Capecitabine + Bevacizumab/Cisplatin + 5-FU or Capecitabine + Placebo	Completed	21 February 2014
RAINFALL	NCT02314117	Gastric + GEJ adenocarcinoma, HER2-negative	1	III	North and South America, Europe, Asia	Cisplatin + 5-FU or Capecitabine + Ramucirumab/Cisplatin + 5-FU or Capecitabine + Placebo	Active, not recruiting	1 March 2019
GAMMA-1	NCT02545504	Gastric + GEJ adenocarcinoma, HER2-negative	1	III	North America, Europe, Australia	Leucovorin + 5-FU + Oxaliplatin + Andecaliximab/Leucovorin + 5-FU + Oxaliplatin	Completed	29 January 2019
FAST	NCT01630083	Oesophageal + gastric + GEJ adenocarcinoma, CLDN18.2 expression	1	II	Europe, Asia	Epirubicin + Oxaliplatin + Capecitabine + IMAB362/Epirubicin + Oxaliplatin + Capecitabine	Completed	11 October 2016
TAGS	NCT02500043	Gastric + GEJ adenocarcinoma	3	III	North America, Europe, Asia	Trifluridine + Tipiracil/Placebo	Completed	21 October 2018
ABSOLUTE	JapicCTI-132059	Gastric adenocarcinoma	2	III	Asia	Paclitaxel/Nab-Paclitaxel	Completed	April, 2017
WJOG 4007	UMIN000001252	Gastric cancer	2	III	Asia	Paclitaxel/Irinotecan	Completed	December, 2013
COUGAR-02	ISRCTN13366390	Gastric + GEJ + oesophageal adenocarcinoma	2	III	Europe	Docetaxel/active symptom control	Completed	15 Janurary 2014
FLOT4	NCT01216644	Gastric + GEJ adenocarcinoma, locally advanced (>T1) and/or nodal positive (N+)	1	II/III	Europe	Epirubicin + Cisplatin + 5-FU or Capecitabine (ECF/ECX)/5-FU + Leucovorin + Oxaliplatin + Docetaxel (FLOT)	Completed	11 May 2019
REGATTA	UMIN000001012	Gastric cancer with a single non-curable factor	1	III	Asia	Oral S-1 + Cisplatin/Gastrectomy followed by oral S-1 + Cisplatin	Completed	March, 2016
RENAISSANCE (AIO-FLOT5)	NCT02578368	Limited metastatic gastric + GEJ adenocarcinoma	1	III	Europe	5-Fluorouracil + Leucovorin + Oxaliplatin + Docetaxel (FLOT)/FLOT + gastrectomy	Recruiting	December, 2021
TOGA	NCT01041404	Gastric + GEJ adenocarcinoma, HER2-positive	1	III	Europe, Asia, Australia, Africa, South America	Cisplatin + 5-FU or Capecitabine/Cisplatin + 5-FU or Capecitabine + Trastuzumab	Completed	28 August 2010
LOGIC	NCT00680901	Oesophageal + gastric + GEJ adenocarcinoma, HER2-positive	1	III	North and South America, Europe, Asia	Capecitabine + Oxaliplatin + Lapatinib/Capecitabine + Oxaliplatin + Placebo	Active, not recruiting	10 February 2016
JACOB	NCT01774786	Gastric + GEJ adenocarcinoma, HER2-positive	1	III	North and South America, Europe, Asia	Cisplatin + 5-FU or Capecitabine + Trastuzumab + Pertuzumab/Cisplatin + 5-FU or Capecitabine + Trastuzumab + Placebo	Active, not recruiting	11 September 2018
GATSBY	NCT01641939	Gastric + GEJ adenocarcinoma, HER2-positive	≥2	II/III	North and South America, Europe, Asia	Trastuzumab emtansine/Docetaxel or Paclitaxel	Terminated	23 March 2017
TyTAN	NCT00486954	Gastric + GEJ adenocarcinoma, HER2-positive	2	III	Asia	Lapatinib plus Paclitaxel/paclitaxel	Completed	1 July 2014
KEYNOTE-811	NCT03615326	Gastric + GEJ adenocarcinoma, HER2-positive	1	III	Unknown	standard of care chemotherapy + Trastuzumab + Pembrolizumab/standard of care chemotherapy + Trastuzumab	Recruiting	Estimated August 2023
KEYNOTE-181	NCT02564263	Oesophageal squamous cell carcinoma + adenocarcinoma	2	III	North America, Europe, Asia	Pembrolizumab/chemotherapy (physician’s choice)	Active, not recruiting	2 July 2019
CHECKMATE-473	NCT02569242	Oesophageal cancer	≥2	III	Unknown	Nivolumab/Docetaxel or Paclitaxel	Unknown	Estimated November 2019
ATTRACTION-3	NCT02569242	Oesophageal squamous cell carcinoma	≥2	III	North America, Europe, Asia	Nivolumab/Docetaxel or Paclitaxel	Unknown	30 September 2019
KEYNOTE-590	NCT03189719	Oesophageal squamous cell carcinoma + adenocarcinoma	1	III	Unknown	Pembrolizumab + Cisplatin + 5-FU/Placebo + Cisplatin + 5-FU	Active, not recruiting	Estimated October 2021
CHECKMATE-648	NCT03143153	Oesophageal squamous cell carcinoma	1	III	Unknown	Cohort 1: Ipilimumab + Nivolumab Cohort 2: Nivolumab + 5-FU + Cisplatin Cohort 3: 5-FU + Cisplatin	Recruiting	Estimated July 2020

* GEJ = gastroesophageal junction.

**Table 2:** Recent and expected approvals of second as well as third and further line therapy strategies in locally advanced and metastatic settings.

Third and Further Lines				Second Line			
Treatment	Trial Name	Tumor Type	Approval	Treatment	Trial Name	Tumor Type	Approval
Nivolumab	ATTRACTION-II	Gastric or GEJ adenocarcinoma	2018: Japan, Taiwan, South Korea	Ramucirumab	REGARD	Gastric or GEJ adenocarcinoma	2014: United States, Europe
Pembrolizumab	KEYNOTE-059	Gastric or GEJ adenocarcinoma, CPS ≥ 1	2017: United States	Ramucirumab + Paclitaxel	RAINBOW	Gastric or GEJ adenocarcinoma	2014: United States, Europe
Apatinib	ANGEL	Gastric or GEJ adenocarcinoma	2014: China	Nab-Paclitaxel	ABSOLUTE	Gastric or GEJ adenocarcinoma	2013: Japan
Trifluridine and Tipiracil	TAGS	Gastric or GEJ adenocarcinoma	2019: United States, Europe	Pembrolizumab	KEYNOTE-181	Oesophageal SCC + adenocarcinoma, CPS ≥ 10	2019: United States
				Nivolumab	ATTRACTION-III	Oesophageal SCC	Not yet approved
				Pembrolizumab	5 KEYNOTE studies	MSI-H (tissue agnostic approval)	2017: United States

## Abbreviations

CI	confidence interval
CPS	combined positive score
EBV	Epstein-Barr Virus
EMA	European Medicines Agency
FDA	U. S. Food and Drug Administration
HR	hazard ratio
MSI	microsatellite instability
MSI-H	microsatellite instability high
OS	overall survival
PD-1	programmed cell death receptor 1
PD-L1	programmed cell death receptor ligand 1
QoL	quality of life
